# Evaluation of the Efficacy and Safety of a Compound of Micronized Flavonoids in Combination With Vitamin C and Extracts of *Centella asiatica*, *Vaccinium myrtillus*, and *Vitis vinifera* for the Reduction of Hemorrhoidal Symptoms in Patients With Grade II and III Hemorrhoidal Disease: A Retrospective Real-Life Study

**DOI:** 10.3389/fphar.2021.773320

**Published:** 2021-12-14

**Authors:** Antonietta G. Gravina, Raffaele Pellegrino, Angela Facchiano, Giovanna Palladino, Carmelina Loguercio, Alessandro Federico

**Affiliations:** Department of Precision Medicine, University of Campania Luigi Vanvitelli, Naples, Italy

**Keywords:** hemorrhoidal disease, flavonoids, vitamin C, *Centella asiatica*, *Vaccinium myrtillus*, *Vitis vinifera*

## Abstract

**Background and Aim:** Several evidences have shown how, in hemorrhoidal disease, phlebotonic flavonoid agents such as quercetin reduce capillary permeability by increasing vascular walls resistance, how rutin and vitamin C have antioxidant properties, and that *Centella asiatica* has reparative properties towards the connective tissue. A retrospective study was designed in order to evaluate the efficacy and safety of a compound consisting of micronized flavonoids in combination with vitamin C and extracts of *C. asiatica*, *Vaccinium myrtillus*, and *Vitis vinifera* for grade II and III hemorrhoidal disease.

**Patients and Methods:** Data of 49 patients, over 18, who were following a free diet regimen, not on therapy with other anti-hemorrhoid agents, treated with a compound consisting of 450 mg of micronized diosmin, 300 mg of *C. asiatica*, 270 mg of micronized hesperidin, 200 mg of *V. vinifera*, 160 mg of vitamin C, 160 mg of *V. myrtillus*, 140 mg of micronized quercetin, and 130 mg of micronized rutin (1 sachet or 2 tablets a day) for 7 days were collected. Hemorrhoid grade according to Goligher’s scale together with anorectal symptoms (edema, prolapse, itching, thrombosis, burning, pain, tenesmus, and bleeding) both before treatment (T0) and after 7 days of therapy (T7) were collected. Primary outcomes were the reduction of at least one degree of hemorrhoids according to Goligher’s scale assessed by proctological examination and compound safety. The secondary outcome was the reduction of anorectal symptoms assessed by questionnaires administered to patients.

**Results:** Forty-four patients (89.8%) presented a reduction in hemorrhoidal grade of at least one grade (*p* < 0.001). No adverse events with the use of the compound were noted. A significant reduction was observed in all anorectal symptoms evaluated (*p* < 0.05). No predictors of response to the compound were identified among the clinical and demographic variables collected.

**Conclusion:** The compound analyzed was effective and safe for patients with grade II and III hemorrhoidal disease according to Goligher’s scale.

## Introduction

Hemorrhoidal disease (HD) is a frequently reported anorectal condition, characterized by distal displacement of the hemorrhoidal cushions and by symptomatic dilation of the anal venous plexus often reported following a persistent high pressure within the hemorrhoidal plexus and which can occur for various reasons (Lohsiriwat, 2012; Godeberge et al., 2021). Overall, several main mechanisms are involved in the pathophysiology of HD, including mechanical injury to the anal cushions, abnormal venous dilatation, venous stasis, vascular thrombosis, tissue inflammation, and degenerative process in the collagen fiber deposition (Lohsiriwat, 2012; Godeberge et al., 2021). Most symptoms result from the enlargement of internal hemorrhoids and are generally associated with constipation, diarrhea, or prolonged defecation, but also with pregnancy and childbirth (Beck et al., 1998). Common symptoms and signs are rectal bleeding, prolapse, itching, and pain (Lohsiriwat, 2012; Perera et al., 2012; Sakr, 2014).

HD is classified, by virtue of clinical severity, into four grades, according to Goligher, depending on whether there is no prolapse (grade I) or, if there was, whether it reduced spontaneously (grade II) or manually (grade III) or was irreducible (grade IV) (Lohsiriwat, 2012; Yamana, 2017; Davis et al., 2018).

Among these active compounds, there are both natural substances (flavonoids, triterpenes, and saponins, etc.), extracted from plants, and synthetic products, like calcium dobesilate (Perera et al., 2012).

The specific mechanism of action of phlebotonic agents, above all if of natural origin, has not been well established; however, their use is associated with the strengthening of blood vessel walls, increase of venous tone, and increase in lymphatic drainage and normalization of capillary permeability (Quijano and Abalos, 2005; Beck, 2011; Lohsiriwat, 2012; Perera et al., 2012; Martinez-Zapata et al., 2016). Furtherly, they showed antioxidant and anti-inflammatory effects, which contribute to vasoprotective actions (Lohsiriwat, 2012; Godeberge et al., 2021). In addition, recent evidence has shown that flavonoids but also vitamin C are involved in the modulation of the anti-senescence gene Sirtuin 1 and with the regulation of nitric acid and that its suppression is associated with cellular mitosis and apoptosis (hajevand-Khazaei et al., 2018; Wei et al., 2014; Shokri Afra et al., 2019; Iside et al., 2020). Quercin also has an immunomodulatory action by inhibiting TNF production in macrophages (Tang et al., 2019).

Several studies evaluated phlebotonics’ effectiveness in hemorrhoid treatment (Alonso-Coello et al., 2006; Aguilar Peralta et al., 2007; Lohsiriwat, 2012; Perera et al., 2012; Giannini et al., 2015; Zagriadskiĭ et al., 2018; Godeberge et al., 2021).

One of the most studied, diosmin has been shown to have capillarotropic, venotropic, and vasotonic properties and to act as a powerful inhibitor of prostaglandins and thromboxane A2 interfering with the activation of leukocytes and of the inflammatory cascade, causing a strong decrease of capillary permeability (monograph; Diosmin, 2004; Bogucka-Kocka et al., 2013).

Other flavonoids act in synergism with diosmin and are efficient for improving microcirculation and vessel health. For example, hesperidin alone and together with other flavonoids reduces permeability and increases capillary resistance. This role has been attributed to its inhibition activity of the hyaluronidase enzyme. Hesperidin anti-inflammatory activity is linked to inhibition of prostaglandins, thromboxane, and the scavenger action of free radicals (Garg et al., 2001).

Quercetin exerts a protective effect on blood vessels thanks to a reduction in capillary permeability and an increase in the resistance of the vessel walls (Chirumbolo, 2012).

Rutin has a protective effect on the walls of blood vessels by acting in the event of telangiectasias, thanks to its neutralizing action of free radicals (Yang et al., 2008). It prevents platelet aggregation and reduces capillary permeability (Sheu et al., 2004).

Several other evidences have showed that a broad number of natural compounds has phlebotonic activity, including anthocyanins from the red grapevine, showing protective properties on arterioles and capillary endothelium, reducing peroxidation of low-density lipoprotein (LDL), and protecting microcirculation from damage caused by diabetes, smoking, and hypertension (Rabe et al., 2011).

In addition, bilberry extract has been shown to be useful for blood circulation (Mastantuono et al., 2016), along with triterpene fraction of *Centella asiatica*, which has peculiar modulating properties on the development of connective tissue. This activity is carried out through an action on fibroblasts and on two essential amino acids for the metabolism of collagen: alanine and proline (Chong et al., 2013). It therefore performs a multi-phase and balanced function on the metabolism of the connective tissue, which results in an improved re-epithelialization and normalization of the perivascular connective tissue, which allows an improvement in the tone and elasticity of the venous wall (Chong and Aziz, 2013).

Vitamin C is a well-known antioxidant, involved in collagen synthesis and in many other cellular biochemical activities. In synergy with bioflavonoids and phlebotonic substances, vitamin C is essential in the maintenance of collagen function, in the strengthening of capillary wall, and for its anti-inflammatory and immunomodulating activities (May et al., 2000). Several research showed that vitamin C protects the endothelium both by stimulating the activity of endothelial nitric oxide synthase (eNOS), resulting in an increase in the production of nitric oxide, and by preventing the oxidation of nitric oxide itself. Nitric oxide in turn is responsible for decreasing the tone of rectal sphincter and relieving pain associated with hemorrhoids, along with improving blood flow (Carr and Frei, 2000).

The aim of this study is to evaluate, using a retrospective design, the efficacy and safety of a compound of micronized flavonoids in combination with vitamin C and extracts of *C. asiatica*, *Vaccinium myrtillus*, and *Vitis vinifera*, administered orally, in patients with grade II and grade III HD.

## Materials and Methods

A retrospective study describing the use of a compound, food supplement, and consisting of micronized flavonoids (diosmin, rutin, quercetin, and hesperidin) in combination with vitamin C and extracts of *C. asiatica*, *V. myrtillus*, and *V. vinifera*, prescribed from 2019 to 2020 in the Hepatogastroenterology Division outpatient unit of the University of Campania “Luigi Vanvitelli” for hemorrhoid disease, was carried out.

### Demographic Variables

Patient demographic data were collected from the outpatient unit database: age, gender, level of exercise, mean number of hours spent in the bathroom, comorbidities, and additional pharmacological therapies.

The level of physical activity was assessed using the International Physical Activity Questionnaires (IPAQ). In the event of a score above 2,520, the subject has been defined as physically active, if between 700 and 2,519 as sufficiently active, and if less than 700 as inactive.

### Clinical Variables

Clinical data, from the outpatient unit database, at two assessment times, at T0 (baseline) and 7 days later (T7), were collected.

The degree of hemorrhoids was assessed on Goligher’s scale. Hemorrhoids were defined as grade I if there were evident prominent hemorrhoidal vessels without prolapse, grade II if there was prolapse with Valsalva and spontaneous reduction, grade III if there was prolapse with Valsalva requiring manual reduction, and grade IV if they were chronically prolapsed with manual reduction ineffective (Davis et al., 2018).

In addition, data on the degree of hemorrhoid edema, hemorrhoid prolapse, and hemorrhoid thrombosis were collected from the same database.

For all three of the above parameters, the assessment was clinical using a three-level score. The grade of edema, prolapse, and thrombosis was defined as grade 0 if absent, grade 1 if resolving, and grade 2 if present.

Clinical data were also collected on patient symptoms: degree of erythema, pain, burning, pruritus, tenesmus, and bleeding.

For all, except bleeding, a four-level scale was used. Symptoms were defined as grade 0 if absent, grade 1 if mild, grade 2 if moderate, and finally grade 3 if severe.

Level of bleeding was assessed as grade 0 if always absent, grade 1 if less than 50% of the evacuations, grade 2 if more than 50% of the evacuations, and grade 3 if always present.

### Inclusion and Exclusion Criteria

A retrospective data collection including patients who met the following criteria was conducted:

1) Clinical diagnosis of HD grade II or III according to Goligher’s scale;
2) Intake for 7 days of a compound in sachets or tablet form already authorized on the market consisting of 450 mg of micronized diosmin, 300 mg of *C. asiatica* dry extract of which 60 mg is total triterpenes, 270 mg of micronized hesperidin, 200 mg of *V. vinifera* dry extract of which 190 mg is proanthocyanidins, 160 mg of vitamin C, 160 mg of *V. myrtillus* dry extract of which 1.6 mg is anthocyanosides, 140 mg of micronized quercetin, and 130 mg of micronized rutin (Flavofort 1500^®^, Merqurio Pharma S.r.l., Naples, Italy). The dose was one sachet or two tablets a day for 7 days;
3) Patients following a free diet regimen;
4) Availability of clinical variables at both time points (T0, baseline; and T7, 7 days after the start of administration);
5) Over 18 years of age;
6) Not on therapy with other anti-hemorrhoid agents;
7) Patients adhering to treatment. Adherence to treatment is routinely assessed using a questionnaire in Hepatogastroenterology Division operating unit for evaluating whether the patient had forgotten to take a few tablets or had thought about stopping a treatment or had stopped or not followed it for at least 90% of the duration. These data were used for studying if enrolled patients were adherent to treatment.

Patients under 18 years of age, with grade I or IV hemorrhoid disease, or patients with clinically significant organic diseases, patients receiving systemic or topical steroid therapy, patients with a prior diagnosis of inflammatory bowel disease or on specific therapy with mesalazine and rifaximin or an enhanced fiber diet were excluded. Patients in whom partial data were obtained or who did not demonstrate adherence to treatment were also excluded.

### Outcomes and Setting

The reduction of at least one grade of HD according to Goligher’s scale was the primary outcome.

Safety was evaluated as an additional primary outcome in relation to the occurrence of patient-reported adverse reactions during and after taking the compound.

Improvement in patient symptoms, as a secondary outcome, was defined as improvement in grade reduction parameters related to erythema, pain, burning, pruritus, tenesmus, bleeding, edema, prolapse, and hemorrhoid thrombosis.

Patients underwent an initial examination in which, by means of an objective examination including rectal exploration, the degree of HD according to Goligher was diagnosed, and clinical symptoms related to anorectal disorders were collected. Patients were prescribed, according to the judgment of the gastroenterologist, different medical therapies, such as a diet rich in fiber and flavonoids, and indication for surgical therapy or the compound object of the study. The same patients were seen again after 7 days in a gastroenterological follow-up visit in which the same procedures were repeated and collected in a clinical database routinely used in the outpatient unit. From the latter, it was possible to extract the data of interest.

### Statistical Analysis

Descriptive statistics were used for the presentation of data. Continuous variables were presented as median (interquartile range). Ordinal variables have been presented as numerosity (percentage of the total) for each degree of freedom. The distribution of the data was evaluated for the choice between parametric and nonparametric tests. Differences in the distribution of the variables in the two assessment times were evaluated by the Wilcoxon signed-rank test. Comparison between the values of the ordinal variables in relation to groups was carried out using the Mann–Whitney U-test. The strength of the correlation between continuous or ordinal variables was analyzed using the Kendall Tau-b test.

To assess the association between demographic parameters and achievement of the primary outcome related to the degree of HD, logistic regression models and risk when identified were expressed as odds ratio (OR) with 95% CI also used.

The accepted level of statistical significance was a *p*-value of less than 0.05 and two-tailed. IBM® SPSS® has been used as software for data analysis. Prism 9® has been used for the processing of graphs.

## Results

### Patient Characteristics

**FIGURE 1:**
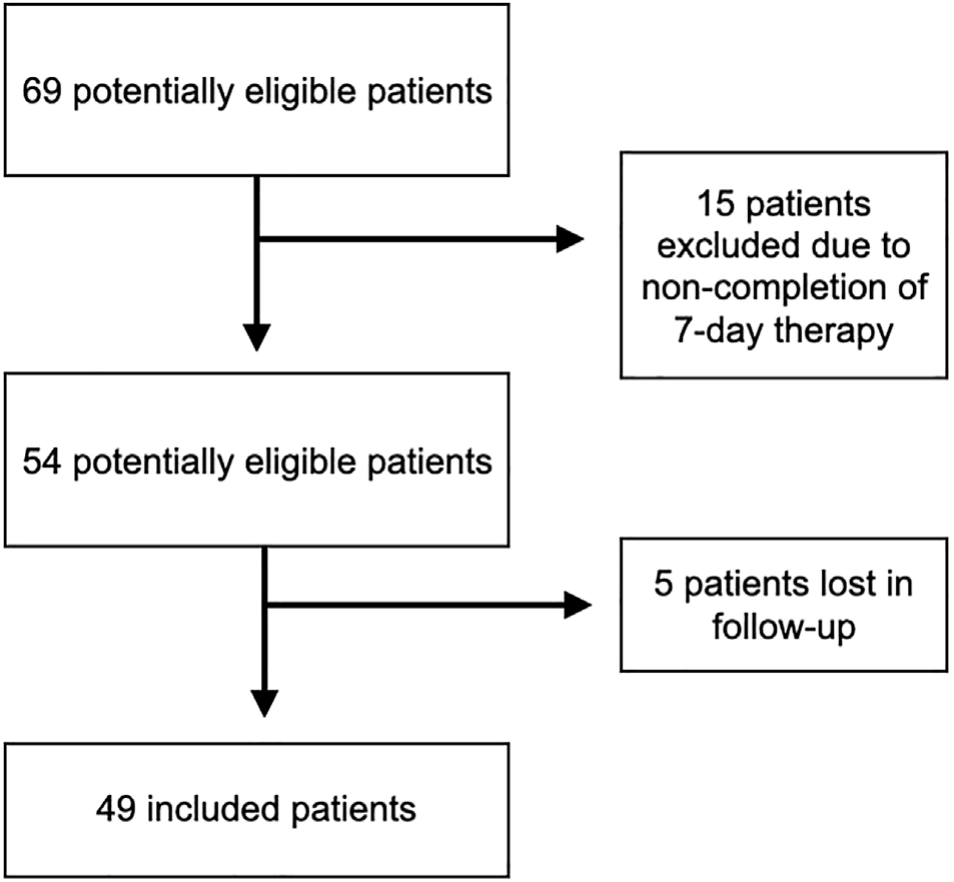
Flowchart describing the selection of patients for inclusion in the study. The number of patients excluded and the reasons for exclusion from the study are shown.

A total of 69 patients were prescribed a compound with a predominant base of micronized bioflavonoids, in combination with vitamin C and extracts of *C. asiatica*, *V. myrtillus*, and *V. vinifera*, from January 2019 to August 2020. Of these, 15 did not complete the 7-day treatment (or had the second visit later than 7 days), while 5 were lost to follow-up (Figure 1). At the end of the study, 49 patients were enrolled. Of these, 30 (61.22%) had grade II HD, while 19 (38.78%) had grade III HD. Twenty (40.8%) patients were male, and 29 (59.2%) were female. In relation to the level of exercise, 19 (38.8%) showed a low level of exercise and 29 (59.2%) moderate level, and finally, only one patient (2%) showed a high level of exercise. The median hours spent in the bathroom was 5 (IQR = 4–8). None of these parameters were significantly different between the two groups (grade II, grade III) as shown in Table 1.

**TABLE 1:** Baseline characteristics of selected patients for the study, divided into two groups according to hemorrhoidal grade (grade II and grade III).

Parameter	Grade II group (*n* 30)	Grade III group (*n* 19)	*p*-Value*
	*N* (%) or Median (IQR)	*N* (%) or Median (IQR)	
Age (years)	43 (33.5–56)	51 (43–62)	0.055
Gender			
Male	12 (40%)	8 (42.1%)	0.885
Female	18 (60%)	11 (57.9%)	
Exercise			
Low	14 (46.7%)	5 (26.3%)	0.222
Moderate	15 (50%)	14 (73.7%)	
High	1 (3.3%)	—	
Hours spent on the WC	5 (4–8)	5 (4–9)	0.827

Note. Data were presented as numerosity (percentage of total) or median (interquartile range (IQR)).

* The *p*-value was obtained comparing if the variable was statistically and differently distributed between the two groups examined.

A total of 13 patients had comorbidities of whom 4 (8.2%) had arterial hypertension, 2 (4.1%) dyslipidemia, and one patient (2%) either urolithiasis or varicosity of the lower limbs, or heart failure or uterine polyps or diabetes. Four patients (8.2%) were taking beta-blockers, 6 (12.2%) renin–angiotensin system inhibitor, 3 (6.1%) cardioaspirin, and 3 (6.1%) statins.

### Outcomes

All 49 patients considered for the study had previously completed the 7-day treatment regimen and assessed parameters at T0 visit and T7 visit.

The primary outcome, reduction of at least one grade of HD according to Goligher’s scale, was achieved by 44 patients (89.8%). Differences in hemorrhoidal grade were, in the range T0–T7, statistically significant (*p* < 0.001).

**FIGURE 2:**
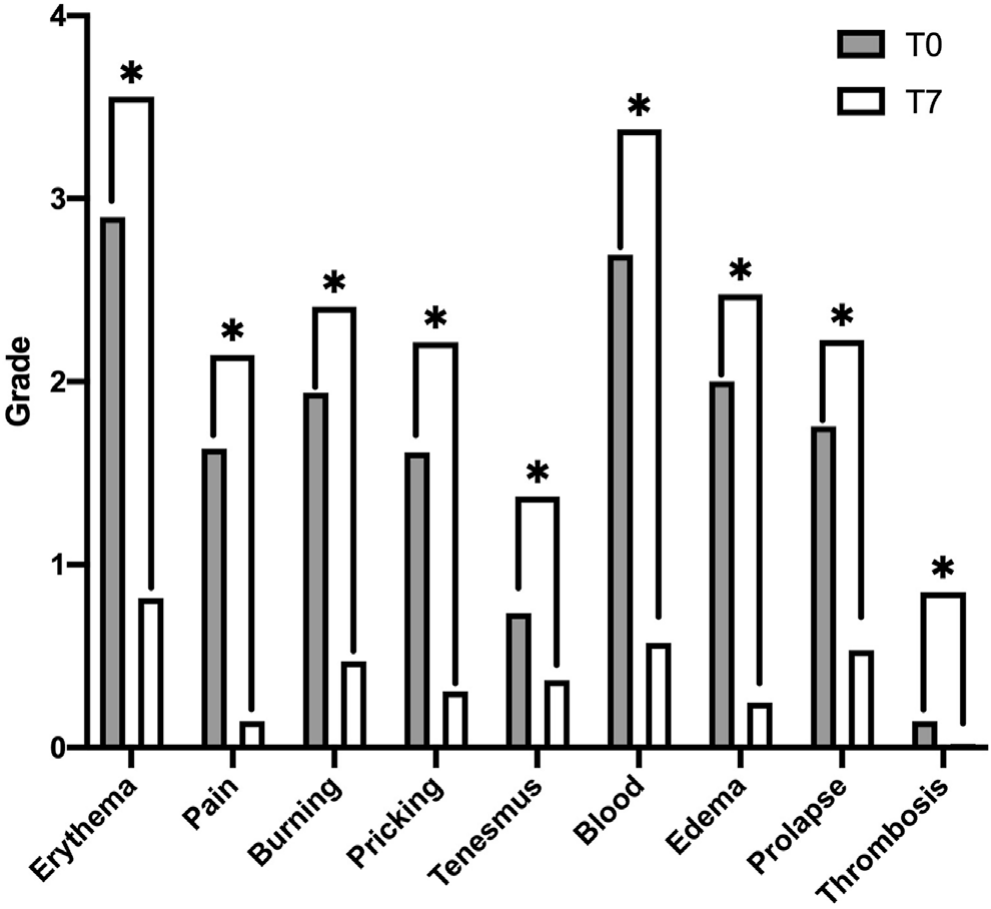
Improvement in hemorrhoidal symptoms, in terms of grade, at the different evaluation times, at baseline (T0), and after 7 days of treatment with the study compound (T7). Data in the histogram are presented as medians. Differences in the distribution of the symptom variable, in the interval T0–T7, and found to be statistically significant are highlighted with “*”.

Table 2 summarizes all clinical variables assessed over the T0–T7 time interval. A general improvement in all parameters analyzed with statistical significance was also observed (Figure 2).

**TABLE 2:** Presentation of changes in hemorrhoidal symptoms at different times of the study, at baseline (T0) and after 7 days of treatment (T7).

Parameter	T0	T7	*p*-Value *	*p*-Value ** (Outcome)	*p*-Value *** (Outcome)
*N* 49	*N* (%)	*N* (%)	T0–T7	T0	T7
Grade hemorrhoid disease					
I	—	31 (63.3%)	**<0.001**	0.119	—
II	30 (61.2%)	18 (36.7%)			
III	19 (38.8%)	—			
Erythema					
Absent	-	12 (24.5%)	**<0.001**	0.761	**0.007**
Mild	1 (2%)	34 (69.4%)			
Moderate	3 (6.1%)	3 (6.1%)			
Severe	45 (91.8%)	-			
Pain					
Absent	21 (42.9%)	45 (91.8%)	**<0.001**	**0.047**	**0.03**
Mild	1 (2%)	1 (2%)			
Moderate	2 (4.1%)	3 (6.1%)			
Severe	25 (51%)	—			
Burning					
Absent	16 (32.7%)	29 (59.2%)	**<0.001**	0.119	0.111
Mild	1 (2%)	17 (34.7%)			
Moderate	2 (4.1%)	3 (6.1%)			
Severe	30 (61.2%)	—			
Pricking					
Absent	20 (40.8%)	39 (79.6%)	**<0.001**	**0.036**	0.051
Mild	3 (6.1%)	7 (14.3%)			
Moderate	2 (4.1%)	1 (2%)			
Severe	24 (49%)	2 (4.1%)			
Tenesmus					
Absent	36 (73.5%)	37 (75.5%)	**0.004**	**<0.001**	**<0.001**
Mild	—	8 (16.3%)			
Moderate	3 (6.1%)	2 (4.1%)			
Severe	10 (20.4%)	2 (4.1%)			
Blood					
Absent	4 (8.2%)	27 (55.1%)	**<0.001**	0.574	0.127
Rare	—	18 (36.7%)			
Frequent	3 (6.1%)	2 (4.1%)			
Always present	42 (85.7%)	2 (4.1%)			
Edema					
Absent	—	38 (77.6%)	**<0.001**	>0.9	0.146
Resolving	—	10 (20.4%)			
Present	49 (100%)	1 (2%)			
Prolapse					
Absent	—	27 (55.1%)	**<0.001**	0.341	**0.001**
Resolving	12 (24.5%)	18 (36.7%)			
Present	37 (75.5%)	4 (8.2%)			
Thrombosis					
Absent	43 (87.8%)	48 (98%)	**0.014**	0.642	0.936
Resolving	5 (10.2%)	1 (2%)			
Present	1 (2%)	—			

Note. Data are presented as numerosity (percentage of total).

* The *p*-value was obtained by assessing whether the distribution of the variables, in the interval T0–T7, was significant.

** The *p*-value was calculated by testing whether, at T0, the variable was associated with statistical strength with the reduction of at least one hemorrhoidal grade according to Goligher (Outcome).

*** The *p*-value was calculated by testing whether, at T7, the variable was associated with statistical strength with the reduction of at least one hemorrhoidal grade according to Goligher (Outcome).

In detail, erythema levels were found to be significantly improved at T7 (*p* < 0.001), and we observed that at T0, the vast majority of patients had severe-grade erythema (45, 91.8%), while at T7, none of the patients presented severe-grade erythema, with the majority of patients (34, 69.4%) having mild-grade erythema and 12 patients (24.5%) having no erythema.

In detail, we observed an improvement in erythema levels at T7 (*p* < 0.001). At T0, it should be noted that the majority of patients presented with severe erythema (45, 91.8%). On evaluation at T7, however, none of the patients presented with severe erythema, and indeed, the majority of patients (34, 69.4%) presented with mild erythema, while 12 patients (24.5%) had no erythema at all.

Pain also improved substantially (*p* < 0.001), whereas at T0, the slightly more than majority of patients (25, 51%) had severe pain; at T7, the substantial majority of patients (45, 91.8%) had no pain.

The same can be said for burning (*p* < 0.001), as the majority of patients at T7 (29,59.2%) had no burning present, starting from a condition at baseline in which 61.2% of patients showed severe burning.

Regarding pruritus, there is again an improvement (*p* < 0.001), with 39 (79.6%) patients at T7 without pruritus; starting from baseline, 24 (49%), 2 (4.1%), and 3 (6.1%) of patients had severe, moderate, and mild pruritus, respectively.

Although at baseline the majority of patients did not present tenesmus (36, 73.5%) while 10 patients (20.4%) presented severe tenesmus, it was observed that, significantly (*p* = 0.004), the number of patients with severe tenesmus decreased to 2 (4.1%) at T7 with redistribution in the less severe grades.

A dramatic reduction in the level of bleeding was observed in the range T0–T7 starting from a baseline condition in which 42 patients (85.7%) had bleeding in every fecal bowel. Specifically, at T7, only 2 patients (4.1%) had bleeding after 7 days of treatment (*p* < 0.001).

Hemorrhoidal edema and prolapse were also parameters that showed improvement at the T0–T7 transition (*p* < 0.001).

Finally, hemorrhoidal thrombosis in resolution or frankly present at baseline (6, 12.2%) decreased at T7, with no patients with frank hemorrhoidal thrombosis, only one patient (2%) with thrombosis still in resolution, and 98% of patients with absent thrombosis (*p* = 0.014). However, it should be noted that the majority of patients (43, 87.8%) at baseline were without hemorrhoidal thrombosis.

It was observed that distribution of data with respect to the primary outcome variable (decrease of at least one degree of Goligher’s hemorrhoid disease) was significantly different with respect to the degree of erythema assessed at T7, degree of pain, degree of pruritus assessed at T0, degree of tenesmus, and finally the degree of prolapse assessed at T0–T7. The remaining clinical parameters showed nonsignificant distributions with respect to the achievement of the primary outcome as shown in Table 2.

At the analysis of the bivariate correlation, achievement of the primary endpoint (reduction of at least one degree of the scale for Goligher’s HD) was confirmed to be strongly correlated with the degree of erythema assessed at T7 (*p* = 0.001), pain assessed at T7 (*p* < 0.001), pruritus assessed at T0 (*p* = 0.022) and T7 (*p* = 0.006), and tenesmus assessed at T0 and T7 (*p* < 0.001) as well as the degree of edema (*p* = 0.043) and prolapse (*p* = 0.001) assessed at T7.

None of the demographic parameters such as age, gender, exercise level, hours spent in the water-closet, medications taken, or comorbidities showed a correlation with outcome attainment as shown in Table 3.

**TABLE 3:** Demographic variable frequencies, expressed as numerosity (percentage of total) or median (interquartile range).

Parameter *N* 49	*N* (%) or median (IQR)	*p*-Value*	OR (95% CI)**	*p*-Value**
Age (years)	47 (37–57.5)	0.911	1.003 (0.933–1.077)	0.9
Gender				
Male	20 (40.8%)	0.987	0.97 (0.140–6.723)	0.9
Female	29 (59.2%)			
Exercise				
Low	19 (38.8%)	0.962	1.196 (0.168–8.53)	0.8
Moderate	29 (59.2%)			
High	1 (2%)			
Hours spent on the WC	5 (4–8)	0.489	0.913 (0.622–1.338)	0.6
Comorbidity				
Hypertension	4 (8.2%)	>0.9	0.496 (0.22–1.119)	0.091
Dyslipidemia	2 (4.1%)			
Urolithiasis	1 (2%)			
Varicosity of the lower limbs	1 (2%)			
Heart failure	1 (2%)			
Uterine polyps	1 (2%)			
Diabetes	3 (6.1%)			
Drugs				
Beta-blockers	4 (8.2%)	>0.9	1.458 (0.936–2.272)	0.096
Renin–angiotensin system inhibitor	6 (12.2%)			
Cardioaspirin	3 (6.1%)			
Statins	3 (6.1%)			
Adverse events	0 (0%)	—	—	—

* *p*-Value was obtained by assessing whether the demographic variables were correlated, with statistical force, and with the reduction of at least one degree of hemorrhoid disease.

** OR with 95% CI and *p*-value were obtained by assessing whether the demographic variables were associated, with statistical force, and with the reduction of at least one degree of hemorrhoid disease.

This result was also confirmed in the logistic regression where none of the demographic parameters were significantly associated with the achievement of the primary outcome related to the reduction of the degree of HD for age (OR = 1.003; 95% CI 0.933–1.077; *p* = 0.9), gender (OR = 0.97; 95% CI 0.14–6.723; *p* = 0.9), level of exercise (OR = 1.196; 95% CI 0.168–8.53; *p* = 0.8), time spent in the bathroom (OR = 0.913; 95% CI 0.622–1.338; *p* = 0.6), medications taken (OR = 1.458; 95% CI 0.936–2.272; *p* = 0.096), or comorbidities (OR = 0.496; 95% CI 0.22–1.119; *p* = 0.091) as shown in Table 3.

By evaluating the visits of patients considered in the study, at time T7, no adverse reaction to the use of the compound had been recorded. In other words, 100% of the patients showed a safe profile of the compound.

All 49 (100%) of the patients included in the study were adherent to the compound for 7 days of therapy. As already mentioned, therapy adherence was assessed by means of a questionnaire. Processing the data from this questionnaire, it was observed that 16 (32.7%) patients expressed difficulty in consistently following the use of one tablet per day of the product, while only 7 (14.3%) patients responded in the questionnaires that they had difficulty remembering how to follow the dosage due to daily commitments. Finally, only 5 (10.2%) patients responded that, while taking the compound, they had thought about stopping it at least once. Ultimately, it was possible to observe that all patients took the compound in full, and generally, the majority of patients took it without difficulty.

## Discussion

This study originates from the observation that heterogeneous classes of compounds have been used for relieving hemorrhoidal symptoms both as a single drug and as combination therapy. The use of a mix of micronized flavonoids in combination with vitamin C and extracts of *C. asiatica*, *V. myrtillus*, and *V. vinifera*, orally administered as a single compound in patients with grade II and III HD, was deepened. Interestingly, results showed that after 7 days of treatment, primary outcome related to the reduction of HD grade was achieved by the majority of patients (about 90%). Statistically significant improvements have been reported in all secondary outcomes evaluated, with frequent symptom disappearance or shifting to mild grade (Table 2).

Numerous studies reported the efficacy of flavonoids and micronized purified flavonoid fraction (MPFF) in HD treatment (Sheikh et al., 2020). Several studies have shown results after 7 days of therapy with MPFF, and by comparing these results with those obtained by this study analysis, overlapping results were noted in terms of efficacy. In particular, in one of them (Cospite, 1994), patients with HD, treated with MPFF (3,000 mg/day for 3 days and 2,000 mg/day for 4 days; *N* = 50) reported pain resolution in 84% of cases and bleeding resolution in 95% of cases. These results are similar to those obtained in this study where pain and bleeding resolution has been achieved in almost 92% of patients.

Another study conducted in patients with HD, treated with MPFF (3,000 mg/day for 4 days and 1,500 mg/day for 3 days; *N* = 49), shows a high percentage of patients with symptom improvement (absent or mild) on day 7: bleeding 98%, pain 94%, pricking 100%, edema 92%, and prolapse 100% (Jiang and Cao, 2006). In this study, the corresponding percentages of patients with symptoms absent or mild on day 7 are bleeding 92%, pain 94%, pricking 94%, edema 98%, and prolapse 92%.

Misra et al. evaluated bleeding control in patients with acute internal hemorrhoids also with an evaluation after 7 days of treatment with MPFF (3,000 mg/day for 4 days and then 2,000 mg/day for 3 days; *N* = 50) showing bleeding cessation in 94% of patients, similar to this work (Misra and Parshad, 2002).

Although these studies showed an overlapping trend of the results, there is a substantial difference between the study setting mentioned above and this study, regarding the intervention, that in the literature, reported studies are mainly made up of high dosage of MPFF, starting with attack therapy of 3,000 mg/day for first 3 or 4 days, followed by 4 or 3 days of lower dose therapy from 2,000 to 1,500 mg/day. The compound of this study consisted of a total of 990 mg of micronized flavonoid (diosmin, hesperidin, quercetin, and rutin) in association with natural dry extracts of 300 mg of *C. asiatica*, 200 mg of *V. vinifera*, 160 mg of *V. myrtillus*, with 160 mg of vitamin C, orally administered as a single compound. All these components are active in the improvement of microcirculation and tone and elasticity of the venous wall (May 2000; Chong and Aziz, 2013; Mastantuono et al., 2016), so it was assumed that efficacy results obtained with the dose here reported could be due to a synergic effect between active ingredients.

Further, the compound in the study, administered as one sachet or two tablets a day, showed high compliance for patients, with 100% of patients adherent to the therapy. It may be possible to speculate that the reduced number of administrations in a day could have improved adherence to therapy. Finally, of note, the compound has been well tolerated, and no adverse events have been reported during treatment.

The encouraging and novel results here reported allow us to suppose that similar combinations of active ingredients can expand the medical therapies recommended in international guidelines for the management of HD and can provide a new therapeutic approach that can limit the need to use more invasive and complicated procedures, such as banding, sclerotherapy, and infrared coagulation (IRC), and currently recommended in case of failure of medical treatment (Davis et al., 2018).

This retrospective study represents a preliminary analysis of the interesting results reported by the compound based on a mix of micronized flavonoids (diosmin, hesperidin, quercetin, and rutin) in combination with vitamin C and extracts of *C. asiatica*, *V. myrtillus*, and *V. vinifera*. This study has some limitations. Firstly, it is a retrospective study, and another limitation is the small number of analyzed patients and the lack of a control group.

Although this analysis can be considered a starting point for subsequent evaluations, certainly new evidence is required from randomized controlled trials (RCTs) evaluating this combination therapy as support therapy for more severe HD grades and/or surgical patients.

In conclusion, this study showed the efficacy and the safety of the compound based on a mix of micronized flavonoids (diosmin, hesperidin, quercetin, and rutin) in combination with vitamin C and extracts of *C. asiatica*, *V. myrtillus*, and *V. vinifera* (Flavofort 1500^®^), evaluated in a retrospective way in patients with grade II and III HD, treated with one sachet or two tablets a day for 7 days.

## Data Availability

The raw data supporting the conclusion of this article will be made available by the authors, without undue reservation.
